# Jewish Perspectives on Porcine Xenotransplantation: Balancing Religious Ethics and Medical Necessity in Israel

**DOI:** 10.1007/s10943-025-02491-4

**Published:** 2025-11-06

**Authors:** Mahdi Tarabeih, Aliza Amiel, Wasef Na’amnih

**Affiliations:** 1 School of Nursing Sciences, The Academic College of Tel-Aviv-Yaffa, 64044 Tel Aviv, Israel; 2 Department of Epidemiology and Preventive Medicine, School of Public Health, Gray Faculty of Medicine, Tel Aviv University, 69978 Tel Aviv, Israel

**Keywords:** Jewish bioethics, Cultural competence, Porcine organs, Xeno-transplantation

## Abstract

Due to the shortage of human organs, this study explores the Jewish perspectives on the permissibility of using pig organs in transplantation. Despite the religious prohibition of consuming pork, Jewish law allows their use for life-saving medical procedures. Our study surveyed 916 Jewish participants, examining their knowledge of religious rulings and their attitudes toward six medical applications of pig-organ transplants. We found that the degree of religious knowledge significantly influences the respondents’ attitudes. Herein, we provide recommendations for enhancing the cultural competence of medical practitioners and improving the informed consent process for Jewish patients for whom xenotransplantation is recommended.

## Introduction

### The Jewish Bioethical Perspective

It is well known that the consumption of pork is forbidden by Torah law. According to the Bible (Tanakh), it is prohibited for all Israelites to eat pork. The Book of Leviticus in the Torah states that pigs “are unclean for you. You shall not eat their flesh, and you shall not touch their carcasses; they are unclean for you” (Leviticus 11:7–8).

Xenotransplantation has been extensively examined in the literature regarding individuals of the Jewish and Muslim faiths. Investigations into religious laws and discussions with scholars have revealed that xenotransplantation does not contravene strict religious laws and is permitted in both Islam and Judaism. It is anticipated that the prevalence of xenotransplantation will increase in the future, especially since pigs are considered the most suitable species for organ transplantation due to their anatomical similarity to humans. There is scarce information available to surgeons regarding the use of animal-derived surgical implants among individuals of different religions. As a result, when surgeons encounter a patient who wishes to avoid animal-derived surgical materials, they are unable to rely on clearly established principles (Loike & Krupka, 2023).

This situation can be challenging for the treating physician; therefore, guidelines are necessary to assist surgeons in addressing religious conflicts that impede medical resolutions. This lack of knowledge has led us to examine this issue, thus assisting surgeons in determining the appropriate course of action when encountering patients who refuse surgical implants based on their religious beliefs (Bokek-Cohen, 2023; Bokek-Cohen & Tarabeih, 2022; Tarabeih et al., 2024). Herein, we focused on the perspectives of Jewish respondents as to the use of animal-derived materials in surgery, based on their religious perceptions.

Judaism mandates the requirement to preserve life, treat disease, alleviate suffering, and ensure that everything possible is done for the patient. Preserving human life is considered a divine commandment; therefore, saving a life is a religious-moral obligation (Rosner, 2005; Steinberg, 2003); hence, all medical decisions must prioritize the patient’s well-being, and the safest and least invasive treatment should always be the preferred option. If consuming food produced from a pig is necessary to save a life, all religious laws are automatically suspended, as Jewish law permits the violation of certain rules to achieve the higher good of protecting a life (Gold & Adams, 2002; Steinberg, 2003). Indeed, Jewish law requires the violation of biblical laws to achieve the higher good of protecting life, and not only regarding the suspension of the rules of kashrut, but Sabbath and festival restrictions as well (Talmud Sanhedrin 74a).

The Jewish bioethical view of xenotransplantation (XTx) relies on the interpretation of Halakhah (Jewish law), derived from the Torah. Halakhah has developed and been expanded over generations through rabbinic discourse recorded in the Mishnah, Talmud, and midrashic texts, and further elaborated upon in medieval and modern codes of Jewish law (e.g., Karo, 1563a, 1563b; Epstein, 1884), as well as in responsa literature across three major branches of Judaism—including Orthodox (Feinstein, 1996), Conservative/Masorti (Roth, 1991; Cherry, 2024), and Reform (Washofsky, 2010).

Until modern times, the layman had limited access to such knowledge; however, today, many Jewish legal subjects, including bioethics, may be investigated by the public interested in the academic literature and following developments in the media.

Pig xenotransplantation offers a promising solution to the critical shortage of donated organs, potentially saving lives and significantly improving the quality of life for patients awaiting transplants (Tarabeih et al., 2024). The Jewish perspective on xenotransplantation, as outlined, emphasizes the central principle of *pikuach nefesh*—the preservation of life—which is paramount in Jewish ethics and values. According to Jewish law (*Halakha*h), saving a human life takes precedence over almost all other religious duties, and this principle permits actions that would otherwise be forbidden. This includes the use of organs and xenografts from pigs, despite the prohibition in the Torah against consuming pork or touching the carcasses of pigs, which are considered ritually unclean. However, handling pig organs when they are not being used for human consumption, especially for medical purposes, is permissible (Loike & Krupka, 2023; Tarabeih et al., 2024).

This does not violate the prohibition against consuming pork, as the prohibition applies to eating the meat and not to medical procedures like transplants. While some concerns arise from the concept of *tumah* (ritual impurity), many rabbinical authorities agree that these concerns do not apply in life-saving situations. Leading Halachic figures, including Rabbis Moshe Feinstein, Mordechai Halperin, and other rabbinic authorities, have ruled that such transplants are allowed when no viable alternative exists and the patient’s life is at risk, based on the Talmud, Tractate Sanhedrin, Chapter 4: “Whoever saves one life, it is as if they have saved an entire world. “Therefore, there is consensus among halakhic authorities in all halakhic movements of Judaism that transplantation of organs from pigs is permitted, indeed encouraged to save a human life.” While some Jews may have cultural reservations about using pig products, Jewish law allows for their use in medical situations. (Feinstein, 1996; Halperin, 2001, cited in Schlesinger Institute, n.d.; Loike & Krupka, 2023).

The ethical considerations in Jewish law also include the humane treatment of animals, prohibiting unnecessary cruelty. The law does not view xenotransplantation as violating these ethical standards as long as the purpose is to save human lives and minimize animal suffering (Tarabeih et al., 2024). Collectively, these obligations and prohibitions form the basis for halakhic discussions and decision-making processes regarding XTx.

When an organ (e.g., a pig heart valve, kidney, or other tissue) is transplanted into a human, the tissue is not destroyed; it remains biologically active and functions as part of the recipient’s body. Over time, the transplanted organ becomes physiologically integrated: blood vessels, nerves, and surrounding tissues grow together with it. Medically, it is considered to have become part of the recipient’s functioning body. Therefore, biologically, the organ retains its structure and function, but it is assimilated into the human system. It no longer functions as “pig tissue” in isolation; it is maintained only through incorporation into the living human (Cooper et al., 2012; Griesemer et al., 2014; Zhang et al., 2022). Contemporary halakhic discussions of pig organ transplantation (Loike & Krupka, 2023; Rosner, 1999) stress that the decisive factor is the *human recipient’s life*. They tend not to treat the transplanted organ as a permanent halakhic “pig object.” Rather, halakha views it functionally as part of the human body, because without integration, it cannot sustain life. Other voices caution that, conceptually, the tissue still originates from a non-kosher animal, but since the purpose is to save life, the prohibition is suspended. In this view, the “pig identity” is not fully erased in theory, but it does not carry practical halakhic weight once transplanted. However, some responsa (Bleich, 2002; Cherry, 2024; Unterman, 1991) argue that once the tissue is transplanted into a living human, it becomes part of the recipient’s body and is no longer subject to the prohibitions of deriving benefit from the dead or from non-kosher sources. In other words, it loses its prior identity and is assimilated.

### Increased Use of Porcine-derived Components for Medical Use

During the COVID-19 epidemic worldwide (March 2019-January 2023), there was a worsening of the waiting periods for organ transplants. However, as of early 2024, the number of people in Israel who were waiting for an organ transplant was 1412; 970 were waiting for a kidney,116 for a liver, 219 for a lung, 97 for a heart, 6 for a heart–lung, and 4 for a kidney-pancreas (Israel Ministry of Health, 2024). Consequently, there is a growing interest in using porcine transplants to help cover this shortfall in human organs to save lives and to improve the quality of life for patients awaiting transplantation.

Pigs are preferred over other mammals for cultivating organs intended for transplants to human recipients because they are easy to raise and reach the size of an equivalent human organ in only six months. For this reason, in recent years, pig heart valve transplants have been routinely performed in humans with arrhythmias, as well as successful experiments in transplanting fetal cells from the pancreas of pigs into type 1 diabetes patients, experiments that suggested an effectiveness in renewing the body’s ability to produce insulin. Moreover, a number of studies have investigated the use of pigskin grafts. In October 2019, American researchers from the Massachusetts General Medical Center reported a breakthrough in this field when they succeeded in transplanting skin tissues from transgenic pigs in burn victims (Elisseeff et al., 2021).

Another study explored pig heart valve implants utilized to regulate blood flow through the heart (Ross et al., 2021). In mesh products, porcine tissue is the most frequently used type of animal tissue, as well as pig DNA, which is used in the pharmaceutical industry. Genetically modified pigs may be used as an alternative for the shortfall of human organs for donation, as they have been associated with an increased survival of recipients (Padilla et al., 2020). Gelatin, usually based on porcine products, is often used in dental and oral surgery grafts (Binti Irfan, 2019; Güngörmüş & Güngörmüş, 2017).

A breakthrough in organ transplantation has been reported by American researchers who successfully transplanted a genetically modified pig heart into a human patient. This transplantation significantly promoted the use of transgenic animal organs and reduced the risk of future rejection of the transplanted organ. The breakthrough came just months after reports of the groundbreaking transplantation of pig kidneys into brain dead human patients at NYU’s Langone Transplant Institute (Wang et al., 2022). Another breakthrough was reported in 2023 relating to the transplantation of a pig’s kidney with only one genetic change and without drugs or experimental devices, which can replace the function of a human kidney. Dr. Robert Montgomery, director of the institute at NYU, who led the procedure, reported that the kidney transplanted in a brain-dead patient continued to function for over a month without being rejected (Locke et al., 2023; Montgomery et al., 2022; Porrett et al., 2022)

### Jewish Legal Discourse on Medical Intervention

The Bible makes many references to the commandment of the practice of healing (see Ex. 21:19; Deut. 4:15;22:2–3). Two prominent medieval halakhic authorities, Moses Maimonides and Yosef Karo, expounded in their codes of law: “The Torah empowers the physician to heal; indeed, this is a religious precept and falls under the mandate to save a life. Should one withhold their medical services, they are deemed akin to one who spills blood” (Karo, 1563a, 1563b). The principle of helping and restoring lost property to its owners is expanded to include restoring their bodies, rooted in the biblical verse, “And you shall restore it to him” (Deuteronomy 22:2). This verse has been interpreted by rabbinic authorities to include the obligation to assist whoever is in need, thus, saving a person who is lost or using one’s physical, intellectual, and financial resources or knowledge to actively offer aid and support for the well-being of one’s neighbor (Maimonides, 1161).

In Judaism, however, the obligation for the individual to seek medical care and treatment is not absolute. Halakhah does permit physicians to administer high-risk or experimental treatments, allowing patients to agree to such treatments under specific circumstances. The specific circumstances of each situation determine whether such medical interventions are justified (Steinberg, 2003). Additionally, medical care that might be deemed futile or lacking in benefit, particularly toward the end of life, may be refused (Mackler, 2012). The duty to preserve human life is emphasized in the verse “So then, choose life” (Deut. 30:19).

Drawing from this and similar teachings, the Rabbis established the general halakhic principle of *pikuach nefesh*, mandating the preservation of human life. According to the Talmud (Sanhedrin 74a- b), saving a human life is of utmost importance in Judaism, taking precedence over nearly all other Jewish laws, thus, one is permitted to violate the laws of the Sabbath and Holy Days to save a life (BTalmud, Sanhedrin 74a). The Talmud brings the words of a rabbinic sage who declares that “God created the cure before He created the disease,” thereby indicating that the laws of nature were created to incorporate the cures for all human illnesses (Loike & Kadish, 2018). *Pikuach nefesh* is rooted in the Torah, particularly Leviticus 18:5; “You shall keep My statutes and My laws, which a person shall do and live by them”. The phrase “and live by them” has been expounded upon by the rabbinic sages to emphasize the principle that the preservation of life is a central value in Jewish law and that the commandments promote life rather than endanger it. Key aspects include the priority given to saving lives over even foundational precepts such as Shabbat and dietary laws. Thus, it permits actions typically prohibited, such as seeking medical assistance or operating medical devices on Shabbat. It also includes communal responsibilities for public health and safety, especially during crises like natural disasters, epidemics, or war. The only commandments that take precedence over *pikuach nefesh* are prohibitions relating to grave transgressions, such as idol worship, sexual misconduct, and murder.

While the Torah explicitly prohibits the consumption of pigs, it does not directly address the raising of pigs. However, rabbinic authorities have extended this prohibition. Jews are traditionally forbidden from raising pigs, a prohibition rooted in rabbinic enactments found in the Talmud (*Bava Kamma* 82b) and codified in later halakhic sources, such as the Shulchan Arukh)Yoreh De’ah 117:1), seemingly reflecting a broader aversion to this animal beyond dietary restrictions. This prohibition is rooted in a historical event cited in the Talmud, prompting the sages to enact such bans (Loike & Krupka, 2023; Babylonian Talmud). It was in this spirit that the Israeli Knesset passed legislation expressing this aversion, beginning with the Pig-Raising Prohibition Law in 1962, which states, “A person shall not raise, keep, or slaughter pigs”, with certain exceptions for local or scientific purposes or display in zoos. The Knesset later went on to pass the Meat and Meat Products Law of 1994, prohibiting all importation of non-kosher meat into Israel (Israel Knesset, 1962; Vered, 2010).

There is limited evidence-based literature relating to the Jewish perspective on using pigs in medical treatments. Jewish medical literature has references to treatments with porcine substances, including fat to cure skin diseases and excessive sweating, bile to treat gynecological problems, dung to stop the bleeding during circumcision, and even drinking urine to melt kidney stones (Shemesh, 2014).

Easterbrook & Madden (2008) explored the views of the three major monotheistic religions regarding the use of porcine-derived implants and found that Jewish religious authorities permitted their use. Interviews of religious authorities of the three religions conducted by Erikkson et al. (2013), reporting on the use of animal-based products for medical treatment, revealed that all of the religious leaders, including the rabbinical authorities, permitted their use for life-saving purposes when no alternate treatments were available. Paris et al. (2018) analyzed Halakhah, exploring Judaism’s view on porcine transplants, and concluded that to save a life, kashrut laws are set aside if treatment involves consuming animal-based products.

Since the late 1600s, halakhic authorities have broadly permitted medical and scientific experimentation on animals (Isserles, Shulchan Aruch EH 5:15; Landau, Noda BiYehuda Kama YD 83; Waldenburg, Tzitz Eliezer 14:68). These practices must fulfill the criteria of being necessary to justify human needs. Furthermore, it is vital to minimize or eliminate pain to the animal as much as possible (Asher, 2017).

### Religious Dilemmas Caused by the Medical Use of Porcine Substances

The advancement of biomedical knowledge and technology has raised crises of conscience for individuals of various religious faiths, who may struggle with the permissibility of availing themselves of certain forms of medical treatment that they perceive may be prohibited by their faith, even when such treatments are permitted by religious law in life-saving contexts (Koshy et al., 2020). Healthcare professionals must be sensitive to these concerns and engage in culturally competent communication when recommending treatments that may raise religious or ethical sensitivities (Sabbouh, 2021). Due to the importance of religion and cultural beliefs to the patient’s recovery process, Greenbaum & Hubbard formulated the concept of “the dominant view” stating that: “… physicians should engage with patients on the patient’s or physician’s substantive religious grounds if the patient cites religious considerations during the process of deliberation” (2019).

One example of these religious considerations occurs when a Jewish patient who is forbidden to consume pork is prescribed a medication, treatment, or medical device based on porcine constituents. Herein, we examine this issue and present the findings of the first empirical study examining the knowledge and attitude of Jews toward using porcine-derived constituents and organs from pigs (Implications of religious and cultural beliefs, 2016).

### The Current Study

Jewish patients may experience distress when their treating physician recommends a medical treatment, such as an organ transplant derived from pigs, due to their religious commitment to the absolute halakhic prohibition against the consumption of pork. Despite the importance of this issue, there is still a lack of empirical data examining the knowledge of the Jewish population regarding the permissibility of using pig organs for medical purposes. There has been no investigation into their stance as to whether religious authorities should permit the use of these transplants.

The current research describes the results of a large-scale research project conducted in Israel, where 76% of the citizens are Jewish (Central Bureau of Statistics, 2024).

We hypothesized that the attitudes of patients toward receiving porcine-based organ transplants are contingent on what they perceive is permitted by their religion. We, therefore, proposed a model explaining the attitude toward porcine-based organ transplantation using sociodemographic variables as well as the respondent’s knowledge as to the permissibility of these treatments.

## Research Objective/Research Questions

The Jewish participants were asked the following:

1. What is your knowledge about the permissibility of the use of porcine-derived organ transplants for medical purposes?
2. What is your attitude toward the permissibility of the use of porcine-derived transplants for lifesaving purposes?

3 Is what you learned about the permissibility of the use of porcine-derived transplants positively correlated with a favorable attitude toward it?

## Methods

### Participants

The target population was Jewish Israeli women and men with varying levels of religious observance, ages, socioeconomic status, and educational levels. The sample consisted of 969 participants. Fifty-three participants did not answer or withdrew before completing the questionnaire, and they were not included in the statistical analysis. Thus, 916 respondents remained. Based on predetermined inclusion criteria, the sample was collected from three different regions of the country (north, center, and south). We used a snowball and convenience sampling method, where each participant helped the researchers find other potential participants through their connections. The questionnaire was posted on social networks for participants who agreed to respond to the online survey.

The only inclusion criterion was age: a minimum age of 18. We examined the level of knowledge, attitudes, and support for the use of pig-derived organs in Jewish patients.

### Measures

The research questionnaire was validated by five experts in the field and translated into English and Hebrew. In addition, a pilot study was conducted on 50 participants, yielding a Cronbach’s alpha score of 0.76, indicating acceptable internal consistency across the knowledge and opinion items in the questionnaire.

The questionnaire comprised three sections. The first examined demographic data such as age, gender, education, religion, level of religiosity, family status, and number of children. The second assessed the level of knowledge about the religious perspective on the use of pig organs for transplantation. In the third section, the participants were asked to express their opinions on whether it is permissible from a religious perspective to donate pig organs for transplantation.

The questionnaire intentionally included different types of organ transplantations, some of which are lifesaving and some less critical. Participants were asked to rate each organ transplant according to its importance and criticality for saving lives. Participants were asked to consider each organ transplant twice: initially to rate, on a 7-point Likert scale, their level of knowledge as to their religious approval of pig organ transplantation based on Jewish tradition and law, with an optional response of “I do not know.” The second time, they were asked to express their opinion, on a 7-point Likert scale, to the extent to which they believed their religion should allow pig organ transplantation. We subsequently constructed two measures by analyzing the responses for 6 medical treatments in terms of the participants’ knowledge as to the religious permissibility, and similarly, the average responses for the permissibility of each medical treatment.

### Ethical Approval

“The researchers had no prior contact with the participants. Informed consent was obtained through a dedicated question at the beginning of the survey, with the research topic “Medical use of organs derived from pigs.” The study received approval from the appropriate institutional review board (Approval No. 2022-1081). Anonymity and discretion were maintained throughout, and respondents could withdraw at any point.”

### Data Analysis

Data cleaning and distribution characteristics, including tests of normality, were examined. The variables were distributed normally and presented by means and standard deviations of each item, and Pearson zero-order correlations between each dyadic set of items. We used the paired-sample *t*-tests for continuous variables to assess the degree of statistical difference between the 6 sets of items. A path analysis model was designed to explain the attitudes toward the use of porcine-derived materials for medical purposes. Statistical analyses were performed using the SPSS-PC (v28) statistical package. The significance level was considered at *P* < .05.

## Results

Overall, 916 Israeli-Jewish participants aged 18–81 years (mean = 51.1, SD = 20.8), 457 (49.9%) were males, 52.5 and 49.1% were academic (university education) and religious participants, respectively. The mean number of children was 3.96, SD = 2.73 (Table 1).

**Table 1:** Demographic descriptive data

Variable	Category	*N*	%	*M*	*SD*	*R*
Gender	Male	457	49.9	-	-	-
	Female	459	50.1	-	-	-
Education	Not academic	435	47.5	-	-	-
	Academic	481	52.5	-	-	-
Religiosity	Religious	450	49.1	-	-	-
	Secular	466	50.9	-	-	-
Marital Status	Not in a relationship	157	17.1	-	-	-
	In a relationship	759	82.9	-	-	-
Age		-	-	51.13	20.78	18–81
Number of children		-	-	3.96	2.73	0–10

N, frequency; %, relative percent; M, mean; SD, standard deviation; R, range

**Fig. 1:**
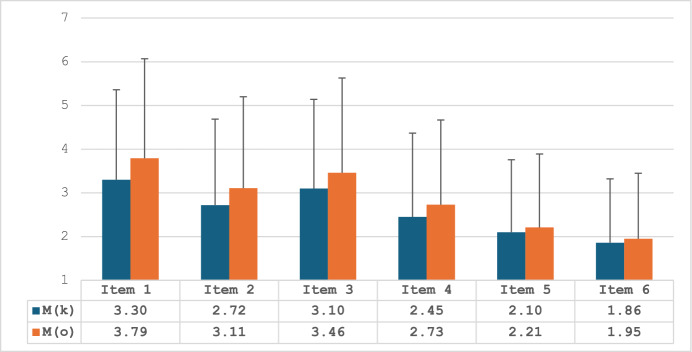
Bar graph for knowledge and opinion item means (standard deviation bars).M = mean. An indication of the letters K and O refer to the different measures, whereas K = knowledge measure, and O = opinion measure

The knowledge and opinion measures consisted of 6 corresponding items as previously mentioned. Table 2 presents the means and standard deviations of each of the items, and the zero-order correlations between each dyadic set of items. Furthermore, paired-sample *t*-tests were used to assess the degree of statistical difference between the 6 sets of items from both measures (for example, item 1 in knowledge vs. item 1 in opinion, item 2 in knowledge vs. item 2 in opinion, and so forth). The means are also depicted in Fig. 1.

**Table 2:** Means, standard deviations, and Pearson zero-order correlations for all knowledge and opinion items

	knowledge items		opinion items				
Item	*M*	*SD*	*M*	*SD*	*r*_p_	*t*-test	*P value*
1. Is it permitted according to the Jewish religion to transplant a heart or a heart valve obtained from a pig into a patient suffering from a heart problem whose life is in danger?	3.30	2.06	3.79	2.28	.92	24.23	< .001
2. Is it permitted according to the Jewish religion to perform a lung transplant into a patient suffering from severe obstructive pulmonary disease using a lung obtained from a pig?	2.72	1.97	3.11	2.09	.95	26.45	< .001
3. Is it permitted according to the Jewish religion to perform a kidney transplant into a patient suffering from severe renal insufficiency using a kidney obtained from a pig?	3.10	2.04	3.46	2.17	.93	22.39	< .001
4. Is it permitted according to the Jewish religion to treat a patient who is in danger of death from pancreatic insufficiency by performing a transplant of pancreas/ pancreatic cells obtained from a pig?	2.45	1.92	2.73	1.94	.95	22.47	< .001
5. Is it permitted according to the Jewish religion to implant into a patient’s knee cartilage obtained from a pig, to replace worn cartilage?	2.10	1.66	2.21	1.68	.96	16.29	< .001
6. Is it permitted according to the Jewish religion to use the skin tissue of a pig to perform a skin graft into a patient with serious burn injuries?	1.86	1.46	1.95	1.50	.96	13.48	< .001

M, mean. SD, standard deviation. R, Pearson zero-order correlation coefficient (all correlations are significant at *P* < *.001*). Generally, the internal consistencies, as measured by Cronbach’s Alpha reliability coefficients, of the knowledge and opinion variables were > .90 for each variable

Table 2 demonstrates very high positive, and significant correlations between the knowledge and opinion items (r_p_ = .92–96) indicating moderate-to-high similarity between the knowledge of the participants on porcine xenotransplantation and their personal opinion. Participants were more assured in their personal opinion than their generic religious knowledge in items 1–6, *P* < .001 (Table 2).

A correlation matrix was gleaned from the data as a basis for further analysis to enhance the understanding of the underlying factors predicting the changes in opinion (Table 3).

**Table 3:** Pearson zero-order correlations

	1	2	3	4	5	6	7	8
1. Age	-							
2. Gender	− .02	-						
3. Education	.06	− .01	-					
4. Religiosity	.02	.01	.01	-				
5. Marital Status	.63^***^	− .13^***^	-.01	-.11^***^	-			
6. Number of children	.83^***^	.11^**^	-.19^***^	-.27^***^	.65^***^	-		
7. Knowledge	.62^***^	− .03	.21^***^	.39^**^	.22^***^	.44^***^	-	
8. Opinion	.75^***^	− .05	.19^***^	.44^***^	.43^***^	.52^***^	.93^***^	-

^*^P < .05, **P < .01, ***P < *.001*

After assessing the preliminary results from Table 3 (for example, moderate-to-high and statistically significant correlations between demographic factors and both the knowledge and the opinion latent factors), path analysis and SEM (structural equation modeling) were employed to test a mediation model, in which the demographic parameters acted as the predictors knowledge was the mediator and opinion, the criterion.

**Fig. 2:**
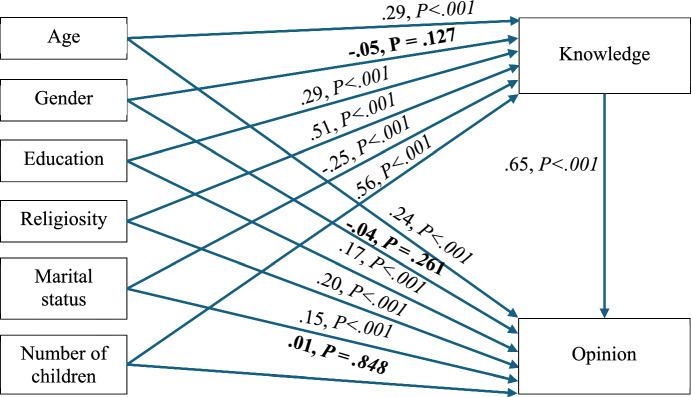
Mediation Model of Knowledge and Opinion Regarding Porcine Xenotransplantation

Mediation analysis, a specific form of path analysis, investigates whether the effect of an independent variable on a dependent variable is transmitted through one or more intermediary (mediator) variables. The goal of mediation analysis is to identify and quantify the extent to which a mediating variable explains the relationship between the independent and dependent variables. Path analysis, by incorporating mediation models, proves to be conducive to better understanding the mechanisms and underlying processes that drive observed associations in the data. In terms of model fit, Fig. 2 had absolute fit to the data (Byrne, 2010): χ^2^ (5) = 11.46, *p* = .043, χ^2^/df = 2.29, SRMR = .03, CFI = .98, GFI = .99, NFI = .99, TLI = .97, RMSEA (90% CI) = .04 (.01–.07), *p-close* = .724. In addition, the path analysis utilized the bootstrapping method to assess the mediation effect (5,000 resamples, 95% bias-corrected confidence interval). Figure 2 suggests that knowledge and opinion are correlated but do not necessarily have a causal relationship due to the predictors (age, gender, education, religiosity, marital status, number of children). The results of the analyses are presented in Tables 4 and 5 and Fig. 2.

**Table 4:** Path analysis results with standardized regression coefficients and difference tests

Path			b	*SE*	*t-test*	*P value*
Age	→	Knowledge	0.29	0.05	6.04	< .001
Gender*	** → **	**Knowledge**	** − 0.05**	0.05	** − 1.01**	**.127**
Education	→	Knowledge	0.29	0.02	12.21	< .001
Religiosity	→	Knowledge	0.51	0.02	20.51	< .001
Marital status	→	Knowledge	** − **0.25	0.03	** − **9.33	< .001
Number of children	→	Knowledge	0.56	0.05	10.92	< .001
Age	→	Opinion	0.24	0.02	14.36	< .001
Gender*	** → **	**Opinion**	** − 0.04**	0.04	** − 0.92**	**.261**
Education	→	Opinion	0.17	0.02	8.50	< .001
Religiosity	→	Opinion	0.20	0.01	19.24	< .001
Marital status	→	Opinion	0.15	0.01	15.26	< .001
Number of children*	** → **	**Opinion**	**0.01**	0.05	**0.19**	**.848**
Knowledge	→	Opinion	0.65	0.01	57.26	< .001

*Bolded results are non-significant

SE, standard error

**Table 5:** Mediation (indirect) effects analyses

Path					Effect	LL	UL	*P value*
Age	→	Knowledge	→	Opinion	.19	.14	.25	< .001
Gender	→	Knowledge	→	Opinion	− .03	− .06	.09	.023
Education	→	Knowledge	→	Opinion	.19	.15	.22	< .001
Religiosity	→	Knowledge	→	Opinion	.33	.29	.37	< .001
Marital status	→	Knowledge	→	Opinion	− .17	− .21	− .13	< .001
Number of children	→	Knowledge	→	Opinion	.36	.29	.42	< .001

Analyses used bootstrapping (95% bias-corrected, 5,000 resamples). Effect, standardized indirect effect (predictor → through mediator → criterion); LL, lower limit of the confidence interval; UL, upper limit of the confidence interval

Table 4 and Fig. 2 indicate that most of the paths/associations were statistically significant: Age positively correlates with knowledge (β = .29, *P* < .001) and opinion (β = .24, *P* < .001) regarding porcine xenotransplantation. Those with an academic education are more assured of their knowledge (β = .29, *P* < .001) and opinion (β = .17, *P* < .001) regarding porcine xenotransplantation than those without an academic education. Religious individuals are more assured of their knowledge (β = .51*, P* < .001) and opinion (β = .20, *P* < .001) regarding porcine xenotransplantation than those who are less religious (for example, secular). Individuals who are not in a relationship are more assured of their knowledge regarding porcine xenotransplantation than those who are in a relationship (β = -.25, *P* < .001). However, Individuals who are in a relationship are more assured of their opinion regarding porcine xenotransplantation than those who are not in a relationship (β = .15, *P* < .001). The number of children positively correlates with knowledge (β = .56, *P* < .001) regarding porcine xenotransplantation. Knowledge positively correlates with opinion (β = .65, *P* < .001) regarding porcine xenotransplantation. However, the individual has more certainty in their generic religious knowledge regarding porcine xenotransplantation when they are certain of their personal opinion.

Moreover, as can be seen in Table 5, most of the indirect effects were significant (for example, the zero is outside the confidence intervals limits), rendering knowledge to be a *partial mediator* between the set of predictors (age, gender, education, religiosity, marital status, number of children) and the criterion (for example, opinion).

In Fig. 2 Mediation Model of Knowledge and Opinion Regarding Porcine Xenotransplantation, the path diagram displays the standardized regression coefficients between demographic predictors, knowledge, and opinion. Figure 2 presents a mediation model showing how sociodemographic variables (age, gender, education, religiosity, marital status, and number of children) predict levels of knowledge and opinion regarding porcine xenotransplantation. Knowledge acts as a significant partial mediator between these predictors and opinion. Key findings include strong positive associations between knowledge and opinion (β = .65, *p* < .001), as well as significant predictive effects of age, education, and religiosity on both knowledge and opinion.

## Discussion

The current study sheds light on organ transplantation with porcine constituents from the perspective of a Jewish patient in Israel committed to halakhic observance, including adherence to kashrut and other ritual laws that may affect medical decision-making.

We report the major findings of the first empirical study examining the knowledge of Jewish individuals in Israel regarding the permissibility of medical treatment using porcine organs. Understandably, not all Jewish people possess the same level of knowledge regarding their religion; thus, a wide range of beliefs and degrees of adherence to religious rules exist. We did not examine actual knowledge as to permissibility, but rather the respondent’s subjectively perceived knowledge of the permissibility.

Our findings indicate that the respondent’s perceived knowledge was limited and often inaccurate, yet nevertheless, remained a strong influence on their attitude toward using porcine organ transplants for medical purposes. The respondents differentiated between the six medical organ transplants presented, expressing more support for approving the use of porcine transplants in life-threatening situations, while opposing transplants that were less life-threatening, such as skin and cartilage transplants.

Most importantly, it was found that patients’ perceived knowledge directly influenced their attitudes regarding religious permissibility. In some cases, Jewish patients refused life-saving treatments involving porcine-derived organs, even when refusal could result in death. These findings highlight the crucial role of culturally sensitive education and religious consultation in such contexts. Proactive engagement—particularly through guidance from knowledgeable rabbis and culturally competent healthcare providers—could empower patients to make informed decisions aligned with both their religious values and medical needs.

### Strengths and Limitations

There is limited literature on healthcare, specifically, for the Jewish people, despite the great importance given to developing and enriching cultural competence; hence, this study is the first to enquire into both the knowledge and the attitudes of Jewish individuals in Israel who practice different levels of religious observance regarding the use of religiously forbidden animals for medical purposes. Consequently, this study contributes to the evolving scholarship of cultural competence as well as medical ethics. Furthermore, the methodology used here could be applied to organ donation and end-of-life care, where patients’ perceptions often diverge from religious rulings.

Our study had some limitations. We relied on a convenience sample-snowball sampling; thus, the generalizability of our findings might be limited. We also used self-reported data on attitudes, which might have resulted in reporting bias. Similar to other studies examining attitudes and worldviews, these views may vary and reflect what respondents expressed at the time of the study.

Our findings suggest that individuals’ attitudes were affected by their knowledge; therefore, some variation among respondents may be attributed to differing opinions in interpreting Jewish dietary laws (Dayton, 2008). Furthermore, some of the respondents were unknowledgeable as to several medical problems described in the study questionnaire. We also found that in most studies using online research questionnaires, there are few respondents over the age of 60 in the sample, thus limiting the sample.

### Interpretation within the Context of the Wider Literature

This research project is timely and relevant in the face of a skyrocketing increase in the use of animal-based components in medicine in the USA and other Western countries (Babos et al., 2021; Dayton, 2008), such as porcine valve implants, cadaver tendons, porcine dermis, corneal transplants, and porcine-based medications. We may soon witness the use of porcine organs for human transplants due to the recent advances in transplant technology (Bokek-Cohen, 2023; Cengiz & Wareham, 2020), thus, making it essential to obtain fully informed consent from certain ethnocultural groups for these products even more compelling (Babos et al., 2021; Bokek-Cohen, 2023; Cengiz & Wareham, 2020; Dayton, 2008; Enoch et al., 2005; Rodger & Blackshaw, 2019).

When participants were asked about their knowledge regarding the permissibility of using various porcine organs according to their religion, ~ 9.03% of the respondents answered “I don’t know” for each of the six different uses of organs presented. Ranked from highest to lowest, the percentages of “I don’t know” answers for each use were: skin transplant − 12% out of 110, cartilage transplant − 11.1% out of 102, pancreas transplant − 10.3% out of 94, kidney transplant − 9.4% out of 86, lung transplant − 8.5% out of 78, and liver transplantation, 7.6% out of 70.

## Conclusions

Halakhah permits XTx that prolong and save human lives, including the use of non-kosher animal organs with genetic modifications. Because the Torah commands to “choose life”, transplants – even from a pig, the ultimate symbol of an unclean animal in Judaism – are permitted.

The study findings showed that most respondents are not knowledgeable about their religion regarding the use of porcine ingredients in medical usage. Further studies should explore the knowledge of Jewish physicians on this issue, as well as the stricter restrictions upheld by all branches of Judaism, including the main Hasidic and ultra-Orthodox groups. When the attending physician is knowledgeable, they may be more sensitive to their obligation to ask the patients about their preferences. Physicians should encourage the patients to seek guidance from their rabbi to obtain competent and fully informed consent to use porcine constituents for life-saving purposes.

There are several faith-based populations where there is a paucity of research regarding the use of animal-derived substances, among them Hindus and Muslims, as well as Seventh-day Adventists (Lawson, 2015). Since the cow is considered sacred by Hindus and Sharia (Islamic law) prohibits the consumption of pigs, similar studies should be conducted to evaluate the knowledge and attitudes of members of these religions toward animal-derived constituents in medical treatment, transplants, and implants.

### New Contribution to the Literature

The most significant contribution of this research is the empirical demonstration of a knowledge gap among Jewish laypersons in Israel regarding the permissibility of porcine xenotransplantation. Jewish patients who are committed to observing kashrut rules may be misinformed about the permissibility of using porcine-derived ingredients in organ transplantation due to the widely accepted knowledge that the Bible labels the pig “an unclean animal”. Physicians should suggest that Jewish patients for whom porcine organs are prescribed take advice from their rabbis, in cooperation with the attending physician or pharmacist, so that optimal medical care can be provided without offending the patient’s religious principles.

The transplant surgeon is committed to saving lives. When a patient is inclined to refuse a procedure based on their religious commitment, it may be effective for the surgeon to consult with the patient’s own religious leader to explore appropriate approaches and options together. This is an integral part of respecting patient autonomy and patient-centered care. Another direction that should be pursued is educating religious leaders about medical issues to help raise their awareness of the possibility of using animal products in surgery. Experience with religious leaders in the past has found that treatment using otherwise prohibited animal components is permissible when no other alternatives exist and the consequence of non-treatment is death. Many religions today offer solutions within the framework of their laws in life-threatening circumstances, particularly in cases where alternative treatments do not exist.

### Recommendations for Rabbis Supporting Jewish Patients Considering Porcine Xenotransplantation

Because studies suggest that Jewish patients may have limited or incorrect knowledge about the permissibility of using pig-derived medical treatments for life-saving purposes, rabbis should provide accessible information about Jewish law and medical ethics to patients and to hospital chaplains [who may themselves not be ordained rabbis] and are often the most immediate resource available to the patient (Ebner et al., 2020). However, for Jewish patients committed to a halakhic lifestyle, guidance from their own rabbi remains central to informed decision-making. This underscores the importance of ensuring that rabbis are well-informed about the medical and halakhic dimensions of procedures such as porcine xenotransplantation.

Awareness of the issue may be raised by lectures or synagogue-based classes, or articles in magazines read by the general Jewish public. In Israel, there are medical hotlines associated with rabbinical authorities that provide information to patients seeking specific advice in medical decision-making, such as the Responsa Project of the Schlesinger Institute for Medical-Halachic Research (Schlesinger Institute, n.d.). A referral to one of these authorities, based on the patient’s specific religious orientation, may help patients make confident decisions based on accurate religious and medical information, which aligns religious values with healthcare priorities. Finally, medical associations should be encouraged to cooperate with rabbinical organizations to educate the community rabbis in the basics of xenotransplantation by sending medical experts as guest lecturers to rabbinical conferences to raise the subject and discuss the halakhic solutions.

We hope that this information will initiate further discussion and thinking on the importance of possessing knowledge of religion in the wider acceptance of clinical XTx among diverse ethnocultural populations.
